# Triptolide, a HSP90 middle domain inhibitor, induces apoptosis in triple manner

**DOI:** 10.18632/oncotarget.24737

**Published:** 2018-04-27

**Authors:** Frederick Zhehao Zhang, Derek Hoi-Hang Ho, Roger Hoi-Fung Wong

**Affiliations:** ^1^ Department of Biology, Hong Kong Baptist University, Hong Kong SAR, Hong Kong; ^2^ Department of Chemistry, University of Hong Kong, Hong Kong SAR, Hong Kong

**Keywords:** triptolide, HSP90, CDC37, apoptosis, cancer

## Abstract

Triptolide (TL) is a potent anti-tumor, anti-inflammatory and immunosuppressive natural compound. Mechanistic studies revealed that TL inhibits tumor growth and triggers programmed cell death. Studies further suggested that TL inhibits heat shock response in cancer cells to induce apoptosis. HSP90β is the major component of heat shock response and is overexpressed in different types of cancers. Given almost all identified HSP90β inhibitors are either N or C-terminal inhibitors, small molecules attacking cysteine(s) in the middle domain might represent a new class of inhibitors. In the current study, we showed that TL inhibits HSP90β in triple manner. Characterization suggests that TL inhibits ATPase activity by preventing ATP binding thus blunts the chaperone activity. TL disrupts HSP90β-CDC37 (co-chaperone) complex through middle domain Cys366 of HSP90β and causes kinase client protein degradation. At the cellular level, the TL-mediated decrease in CDK4 protein levels in HeLa cells causes reduced phosphorylation of Rb resulting in cell cycle arrest at the G1 phase. Furthermore, our results demonstrated that TL triggers programmed cell death in an HSP90β-dependent manner as knockdown of HSP90β further sensitized TL-mediated cell cycle arrest and apoptotic effect. Surprisingly, our data showed that TL is the first drug to be reported to induce site-specific phosphorylation of HSP90β to drive apoptosome formation in the early phase of the treatment.

In summary, our study established that TL is a novel middle domain HSP90β inhibitor with bi-phasic multi-mechanistic inhibition. The unique regulatory mechanism of TL on HSP90β makes it an effective inhibitor.

## INTRODUCTION

Molecular chaperones facilitate proper protein folding and allow protein complex to assemble effectively [1]. The function of molecular chaperones is critical to cellular survivals. However, molecular chaperones are double-edge swords as they prevent oncoproteins from misfolding and degradation in cancer cells [2]. Chaperones are highly expressed and heat shock responses are constitutively activated in cancer cells. Different chaperones, such as HSP90β, HSP70 and HSP27, were reported to have oncogenic functions [3]. HSP90β is the major component of heat shock response and is overexpressed in different types of cancers. HSP90β is a critical factor for cancer cell survival and oncogene addiction. Homodimeric HSP90β has 3 distinct domains: C-terminal, middle and N-terminal domains. C-terminal domain of HSP90β is responsible for dimerization and tetratricopeptide repeats (TPR) co-chaperone recognition. The middle domain interacts with most of the non-TPR co-chaperones and client proteins. And ATP-binding pocket is located in the N-terminal domain [4–6]. The conformation and chaperone activity of HSP90β is regulated by ATP and co-chaperones including HOP, CDC37, p23 and Aha1. HSP90β and its co-chaperones work in concert to regulate the conformation and activity of a large variety of signalling molecules, transcription factors and cytoskeletons in response to different cellular stresses [7].

A number of oncogenic client proteins are chaperoned by HSP90β, such as Raf-1, IKK-1/2 and AKT-1, which are involved in different signal transduction pathways during cancer development [8]. Dysfunction of HSP90β by HSP90β inhibitors leads to degradation of its client proteins and triggers programmed cell death [9, 10]. Several studies demonstrated that HSP90β complexes in tumor cells have much higher affinity for ATP and HSP90β inhibitors when compared to those in normal cells, therefore suggesting the therapeutic potential of HSP90β inhibitors for killing cancer cells due to their high selectivity [11–13]. The chaperone activity of HSP90β is required for associating with client proteins and co-chaperones. CDC37 is a non-TPR co-chaperone of HSP90β which was first identified as a cell cycle regulator in budding yeast. Study showed that CDC37 is responsible for recruitment of premature kinase client proteins to HSP90β [14, 15]. CDC37 deficiency reduces HSP90β-CDC37 dependent kinases and increases drug sensitivity of HSP90β inhibitors [16]. On the other hand, recent study showed that CDC37 slows down HSP90β dimer twisting rate without affecting nucleotide accessibility to its binding site(s) [17]. Further evidences suggest CDC37 binds to both N-terminus and middle-domain of HSP90β and restricts its mobility [18]. Most of the HSP90β inhibitors have no effect on the interaction of HSP90β-CDC37 complexes. Natural products Celastrol (CEL) and withaferin A (WA) are the only two identified HSP90β inhibitors that destabilize HSP90β-CDC37 complexes through unknown mechanism [19, 20]. Given almost all identified inhibitors such as ganetespib are either N or C-terminal inhibitors [21], small molecules attacking cysteine(s) in the middle domain may represent a new class of inhibitors that is distinct from those targeting either the N-terminal ATP-binding pocket or the C-terminal dimerization domain of HSP90β [22].

Triptolide (TL), isolated from *Tripeterygium wilfordii* Hook f. (TwHf), exhibits diversified biological activities including anti-proliferation, cytotoxicity, immune modulation and anti-inflammation [23]. Mechanistic studies revealed that TL inhibits tumor growth and triggers programmed cell death through both p53 dependent and independent death-receptor signaling pathway and mitochondria-mediated apoptotic pathway [24–26]. Studies suggested that TL inhibits HSP70 activity in pancreatic cancer cells to induce apoptosis by suppression of HSF-1 [27]. In the present study, we have shown that TL inhibits HSP90β ATPase activity and attacks middle domain cysteine to block the interaction between HSP90β and CDC37. Our further characterization has revealed that TL as a novel inhibitor of HSP90β induces apoptosis in triple manner. We demonstrated that TL has a unique biphasic inactivation of HSP90β through cysteine modification and post-transitional modification of HSP90β in a time dependent manner. To date, TL is the first drug reported to have an effect on the site-specific phosphorylation of HSP90β that is critical for apoptosome formation. TL, a novel middle domain inhibitor, inhibits the ATPase and chaperone activity of HSP90β to induce apoptosis.

## RESULTS

### Triptolide inhibits ATPase activity of HSP90β and disrupts its chaperone activity

**Figure 1:**
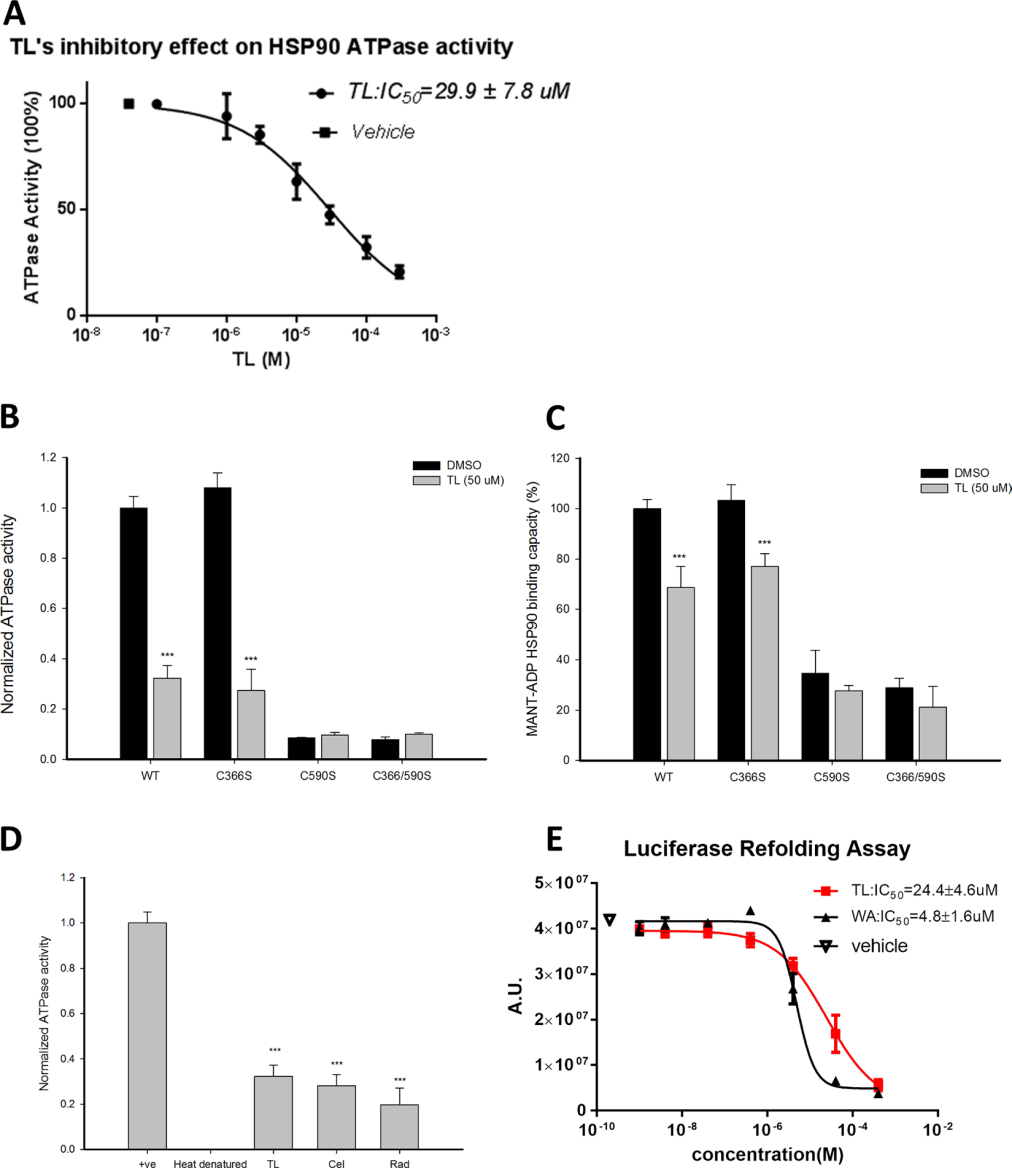
Triptolide inhibits ATPase activity of HSP90β and disrupts its chaperone activity (**A**) TL inhibits ATPase activity of HSP90β in a dosage-dependent manner. Colorimetric ATPase activity assay was performed with different concentrations of TL and DMSO as vehicle control. TL's IC_50_ on the ATPase activity of HSP90β was determined from three independent experiments. Data are represented as mean ± SD (*n* = 3). (**B**) Colorimetric ATPase activity assay was performed with different HSP90β mutants. Recombinant HSP90β (WT and different mutants) was incubated with 50 μM TL at 4°C for 3 hrs, and reaction mixture from assay kit was added and incubated at 37°C for another 1 hr. Absorbance at 650 nM was measured and result from vehicle (DMSO)-treated wild-type HSP90β group was normalized as 1. ^***^P < 0.001 DMSO versus TL. Data are represented as mean ± SD (*n* = 3). (**C**) MANT-ADP binding assay with different HSP90β mutants in the presence of TL. Recombinant HSP90β (WT and different mutants) was incubated with 50 μM TL at 4°C for 3 hrs. MANT-ADP was then added to the reaction mixture. The reaction mixture was incubated for additional 1 hour at 37°C and analyzed with excitation at 370 nM and emission at 465 nM. Result from vehicle (DMSO)-treated wild-type HSP90β group was set as 100% binding capacity. ^***^P < 0.001 DMSO versus TL. Data are represented as mean ± SD (*n* = 3). (**D**) ATPase activity of HSP90β upon treatment of TL and different inhibitors. Recombinant HSP90β was incubated with 50 μM TL, CEL and RAD. Heat-denatured recombinant HSP90β was served as positive control. Colorimetric ATPase activity assay was performed as described above. ^***^P < 0.001 different inhibitors versus heat denatured group. Data are represented as mean ± SD (*n* = 3). (**E**) TL inhibits chaperone activity of HSP90β. HSP90β chaperone activity in the presence of TL and WA at different concentrations. IC_50_ was determined from three independent experiments by measuring the amount of refold heat-denatured luciferase in rabbit reticulocyte lysate. To perform heat denaturing, luciferase was heated at 41°C for 10 min. Rabbit reticulocyte lysate was added to heat-denatured luciferase in the presence of TL and WA in different concentrations, and incubated at 4°C for 3 hrs. Luciferase assay buffer were then added and incubated at room temperature for 5 mins. Luminescence signal was measured with integration time of 400 ms. IC_50_ was determined from three independent experiments. Data are represented as mean ± SD (*n* = 3).

Since TL was reported to trigger cellular apoptosis through HSP70 inhibition, we would like to investigate if the apoptotic effect of TL was mediated by HSP90β, the critical molecular chaperone with anti-apoptotic activities. ATPase activity is crucial to the function of HSP90β chaperone activity as HSP90β dimerization and co-chaperone recruitment are facilitated by its activity. We first measured the ATPase activity of HSP90β in the presence of TL at various concentrations. A dose-dependent inhibition of HSP90β ATPase by TL was observed by incubating 1 μM of HSP90β with increasing concentrations of TL for three hours, of which IC_50_ of TL on HSP90β ATPase activity is 29.9 μM (Figure 1A). Studies have shown that the epoxide group of triptolide, which is the reactive electrophile for thiols, is involved in the covalent modification of cysteines [28]. Out of the 6 cysteines in HSP90β's middle domain and C-terminal, 2 of those at 366 and 590 are exposed and are critical for its ATPase activity and dimerization respectively [29]. We next asked whether these cysteines are required for TL to mediate the inhibition on the ATPase activity by examining the effect of TL on ATPase activity of different HSP90β mutants. In Figure 1B, HSP90β^C366S^ showed similar ATPase activity when compared to HSP90β^WT^ indicating cys366 is not a crucial site to modulate its ATPase activity. Furthermore, TL exhibited a similar inhibitory effect on both HSP90β^WT^ and HSP90β^C366S^ suggesting that TL suppresses the ATPase activity of HSP90β independent of Cys366. Unlike HSP90β^C366S^, in the absence of TL, both HSP90β^C590S^ and double mutant HSP90β^C366/590S^ only showed marginal ATPase activity as Ruiz et al [30] reported that cysteine 590 is a regulatory site for its ATPase activity. Incubation with or without TL with either HSP90β^C590S^ or HSP90β^C366/590S^ did not have any further effect on the ATPase activity suggesting that TL-mediated suppression of the activity could be further blunted by modifying Cys590.

Further validating our hypothesis, MANT-ADP releasing assay was employed to investigate the effect of TL on the nucleotide-HSP90β interaction. Similar to ATPase activity, Figure 1C showed that mutation of cysteine 366 had no effect on TL's inhibition of MANT-ADP interacting with ATP binding pocket. However, the interaction between either HSP90β^C590S^ or HSP90β^C366/590S^ and MANT-ADP was significantly blocked regardless of treatment of TL. Consistent with TL's effect on ATPase activity of HSP90β, TL weakened the interaction between nucleotides and HSP90β^WT^ or HSP90β^C366S^ while it had no effect on neither HSP90β^C590S^ nor HSP90β^C366/590S^. Given cys590 is the regulatory site for ATPase activity, the likelihood of TL inhibiting ATPase activity of HSP90β through modification of cys590 could not be ruled out. We next compared the inhibitory effect of TL alongside with other well characterized HSP90β inhibitors. Figure 1D showed similar inhibitory effect on ATPase activity of HSP90β among 50 μM of TL, CEL and RAD treatment.

We then sought the functional consequence of TL-mediated inhibition of ATPase activity of HSP90β on its chaperone activity. Luciferase refolding assay was conducted in HSP90β-rich rabbit reticulocyte lysate [31]. Incubation of TL in rabbit reticulocyte lysate prohibited HSP90β from restoring heat-denatured luciferase activity in a dose dependent manner with an apparent IC_50_ of 24.4 μM (Figure 1E). WA served as a positive control and its apparent IC_50_ of 4.8 μM which matched the reported value. Notably, IC_50_ of TL in both luciferase refolding assay and ATPase assay are in the similar range suggesting the shutdown of HSP90β chaperone machinery is dependent on its ATPase activity.

### Triptolide attacks the middle domain to detablize the HSP90β-CDC37 interaction

**Figure 2:**
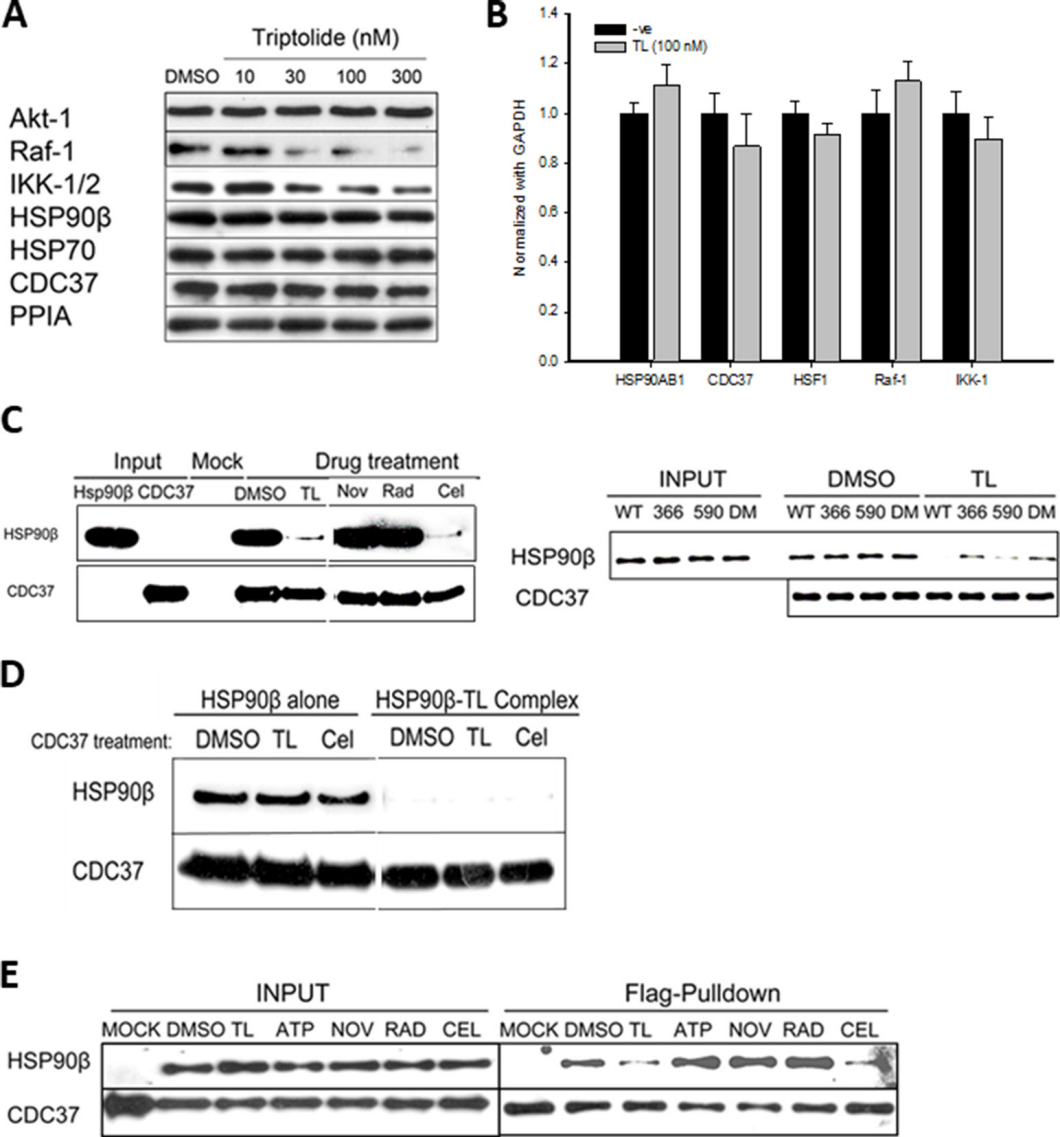
Triptolide destablizes HSP90β-CDC37 complexes and causes selective client protein degradation (**A**) TL selectively decreases levels of HSP90β client proteins in HeLa cells in a dosage-dependent manner. Western blotting of HSP90β client proteins and co-chaperones 24 hours after TL treatment (10, 30, 100 and 300 nM) in HeLa cells. (**B**) TL does not regulate transcription levels of HSP90β's client proteins and co-chaperones. RT-PCR analysis of HSP90β client proteins and co-chaperones 24 hours after TL treatment in HeLa cells. Expression level of client proteins and co-chaperones was normalized with GAPDH. (**C**) TL and CEL disrupts the interaction between HSP90β and CDC37 *in vitro*. Recombinant HSP90β were incubated with His-tagged CDC37 in the presence of TL and different inhibitors (NOV, RAD and CEL) *in vitro* prior to Ni-NTA pulldown. Western blotting analysis of eluates using anti-HSP90β and anti-CDC37. Results represented from three independent studies were shown (left). Different HSP90β mutants were incubated with His-tagged CDC37 in the presence of TL *in vitro* prior to CDC37 immunoprecipitation. Western blotting analysis of *in vitro* immunoprecipitated and representative image from three independent experiment was shown (right). (**D**) TL dissociates HSP90β-CDC37 complex by binding to HSP90β alone. CDC37 was pretreated with various drugs and unbound drugs were washed out, followed by incubation with HSP90β and HSP90β/TL complex (HSP90β pre-treated with TL) respectively. CDC37 immunoprecipitation was performed and western blotting analysis of *in vitro* immunoprecipitated mixture was shown and image was represented from three independent experiment. (**E**) TL and CEL disrupts the interaction between HSP90β and CDC37 in cultured cells. Co-immunoprecipitation of flag-tagged CDC37 and strep-tagged HSP90β by anti-FLAG antibodies upon 6 hrs’ pre-treatment of TL and different inhibitors in HEK-293T cells and immunoblotted with streptavidin-HRP (HSP90β) and anti-flag antibody (CDC37). Image was represented from three independent experiment.

Given that TL inhibits intrinsic ATPase activity and chaperone activity of HSP90β *in vitro*, we next examined whether TL could inhibit the chaperone activity in cultured cells. TL has been reported to inhibit the proliferation of all 60-NCI cancer cell lines including HeLa with an IC50 in the nanomolar range [32, 33], and HeLa cells have been adopted to study the anti-proliferative effect of TL [28, 34]. Treating HeLa cells with TL at 30 nM or higher concentrations drastically reduced protein levels of Raf-1, a client protein of HSP90β (Figure 2A). We also detected that Raf-1 was highly ubiquinated in the presence of TL (Supplementary Figure 1). In contrast, no significant change was observed in protein levels of other HSP90β client proteins such as AKT-1 in response to TL treatment. Abundance of HSP90β and co-chaperone CDC37, however, was unchanged in the presence of TL. These results suggest that TL impairs HSP90β machinery without affecting protein levels of the chaperone complex (Figure 2A). It has been reported that TL interacts with XPB to inhibit universal transcription, thus we tested whether TL could specifically down-regulate mRNA levels of HSP90β client proteins and co-chaperones. Surprisingly, TL treatment at 100 nM for 24 hours did not change the mRNA levels of HSP90β, its client proteins, co-chaperone and cofactors (Figure 2B). These results indicate that impairment of HSP90β chaperone machinery by TL is independent of transcriptional and post-translational regulation in HeLa cells.

Intrigued by study demonstrated that function of Raf-1 requires both active HSP90β and its co-chaperone CDC37 [35] and our observed reduction of Raf-1, we next investigated whether TL could have any direct effect on the HSP90β-CDC37 complex as TL does not affect either protein or gene expression of CDC37 as shown previously. His-tagged CDC37 was incubated with HSP90β prior to *in-vitro* His pull-down assay in the presence of different known HSP90β inhibitors including novobiocin (NOV), radicicol (RAD) and celastrol (CEL). While a strong interaction was detected between HSP90β and CDC37 in the absence of inhibitors, TL and CEL treatment disrupted the HSP90β-CDC37 complex (Figure 2C, left panel), and HSP90β inhibitors NOV and RAD did not have obvious effect on the HSP90β-CDC37 interaction. Consistent with published studies, a significantly reduced amount of wild type HSP90β was co-pulled down by CDC37 when pretreated with CEL compared to control group. To dissect the exact mechanism employed by TL to disrupt the HSP90β-CDC37 complex, different HSP90β mutants carrying the exposed cysteines were incubated with CDC37 in the presence or absence of TL (Figure 2C, right panel). In the absence of TL, comparable amount of wild-type HSP90β and HSP90β mutants (C366S and C590S) could be co-pulled down by CDC37 indicating that mutation of either cysteine 366 or 590 has no direct effect on the interaction between HSP90β and CDC37. However, for the mutant HSP90β^C366S^, CDC37 was able to capture similar amounts of HSP90β even in the presence of TL indicating that TL disrupts HSP90β-CDC37 complexes through Cys366. Taken together, TL disrupts the interaction between CDC37 and HSP90β mainly through modification of middle domain cysteine of HSP90β despite that the site has no effect on the interaction itself.

In order to test whether TL could have any effect on CDC37 to disrupt the interaction, we next investigated the interaction between drug pretreated CDC37 and HSP90β. Results showed that HSP90β alone could pull down CDC37 regardless pretreatment of TL or CEL with CDC37. However, only TL treated HSP90β destabilized the HSP90β-CDC37 complexes among all experimental groups (CDC37 upon control, TL and CEL pretreatment) (Figure 2D). Non-reducing gel WB also revealed that TL, CEL and WA caused no significant change in oligomeric status of CDC37 (Supplementary Figure 2A). Given TL has no effect on CDC37, TL effects on HSP90β to disrupt its interaction with CDC37.

We further examined the effect of TL on the disruption of the HSP90β-CDC37 interaction in cultured cells. In the presence of TL or CEL, HSP90β could not be co-purified with FLAG-tagged CDC37 in HEK-293T cells. In contrast, ATP, NOV and RAD treatments had no effect on the interaction between the two as indicated by the positive outcome of the co-purification of HSP90β with CDC37 (Figure 2E). Time course study and dose-dependent experiment of TL in HEK-293T cells also revealed that TL disrupted HSP90β-CDC37 complex in both time dependent and dosage dependent manners (Supplementary Figure 2B). Overall, these data showed that TL disrupts the interaction between HSP90β and CDC37 in cultured cells.

### Triptolide inhibits HSP90β to arrest cell cycle and triggers programmed cell death

**Figure 3:**
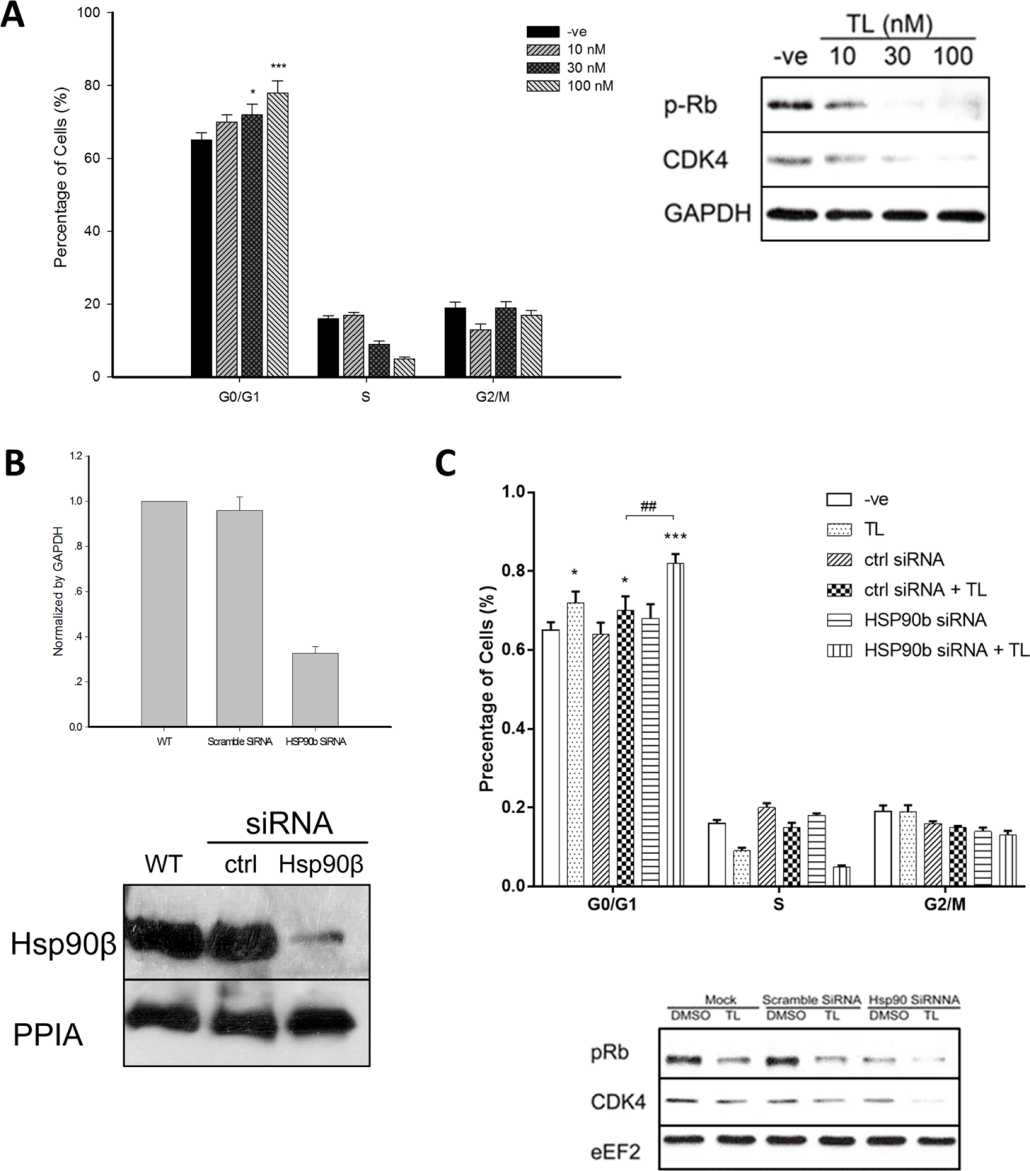
Triptolide arrests HeLa cell cycle through HSP90β (**A**) TL induces cell cycle arrest of HeLa at G1/G0 phase in a dosage dependent manner. Flow cytometry analysis (left) of cell cycle profiles of HeLa cells upon 24 hrs’ TL treatment (10, 30 and 100 nM). ^*^P < 0.05; ^***^P < 0.001 compared with vehicle control. Data are represented as mean ± SD (*n* = 3). Western blot analysis (right) of cell cycle related proteins in HeLa cells at various TL concentrations for 24 hrs. Representative image from three independent experiment was shown. (**B**) Knockdown efficiency of HSP90β by siRNA. mRNA (upper) and protein (lower) level of HSP90β in HeLa cells upon transfection either with control siRNA or HSP90β siRNA was measured. Data are represented as mean ± SD (*n* = 3). (**C**) Knockdown of HSP90β sensitizes TL's cell cycle arrest effect on HeLa cells. Flow cytometry analysis (upper) of cell cycle profiles of HeLa cells transfected either with control siRNA or HSP90β siRNA in the presence and absence of TL for 24 hrs. ^*^P < 0.05; ^***^ *P* < 0.001 vehicle versus TL. ^##^*P* < 0.01 ctrl siRNA+TL versus HSP90β siRNA+TL. Data are presented as mean ± SD (*n* = 3). Western blot analysis (lower) of cell cycle-related proteins in HeLa cells transfected either with control siRNA or HSP90β siRNA in the presence and absence of TL for 24 hrs. Representative image from three independent experiment was shown.

Since CDC37 controls cell cycle division, we next explored TL's effect on cell cycle profiling in cultured cells. Flow cytometry analysis revealed that TL could result in significant cell cycle arrest of HeLa cells at G1 phase in a dosage dependent manner which was indicated by a significant accumulation of cells at G0/G1 phase and an obvious reduction of cells at S phase (Figure 3A, left panel). CDK4, a client protein of HSP90β-CDC37 complexes, is responsible for cell cycle progression from G1 to S phase. We then asked whether TL-mediated disruption of HSP90β-CDC37 complexes could induce cell cycle arrest through decreasing CDK4 protein levels. Western blotting showed that treatment of TL at 30 nM and 100 nM in HeLa cells substantially reduced CDK4 protein levels. Phosphorylation of Rb, the immediate downstream target of CDK4 to regulate cell cycle check-point at late G1 phase, was also reduced by TL in a dosage dependent manner (Figure 3A, right panel).

In order to examine the requisite role of HSP90β in TL-mediated cell cycle arrest, we tested the biological consequences of TL's effect on HSP90β-deficient HeLa cells. Knockdown of HSP90β using corresponding siRNA was efficiently conducted and expression at both transcriptional level (70% reduction) and translational level (90% reduction) was reduced (Figure 3B). Similar to our previous results, flow cytometry analysis revealed that knockdown of HSP90β marginally accumulated HeLa cells in G1/G0 phase when compared with control siRNA transfected cells. TL treatment significantly further increased HeLa cell arrested in G1/G0 phase in HSP90β deficient cells. In contrast, there was no difference between percentages of cells being arrested in G1/G0 phase in TL-treated control siRNA transfected cells and in TL-treated non-transected cells. Importantly, TL-treated HSP90β-knockdown cells showed significant and substantial accumulation in G1/G0 phase when compared with TL-treated control siRNA transfected cells (*p* < 0.01) indicating the importance of HSP90β on the cytotoxic effect of TL (Figure 3C, upper panel). In line with the flow cytometry data, knockdown of HSP90β alone decreased CDK4 protein expression and phosphorylation levels of Rb while TL treatment potentiated the reduction (Figure 3C, lower panel). Taken together, knockdown of HSP90β sensitized TL's cell cycle arresting effect in G0/G1 phase in HeLa cells suggesting TL-induced cell cycle arrest in HeLa cells is HSP90β dependent.

**Figure 4:**
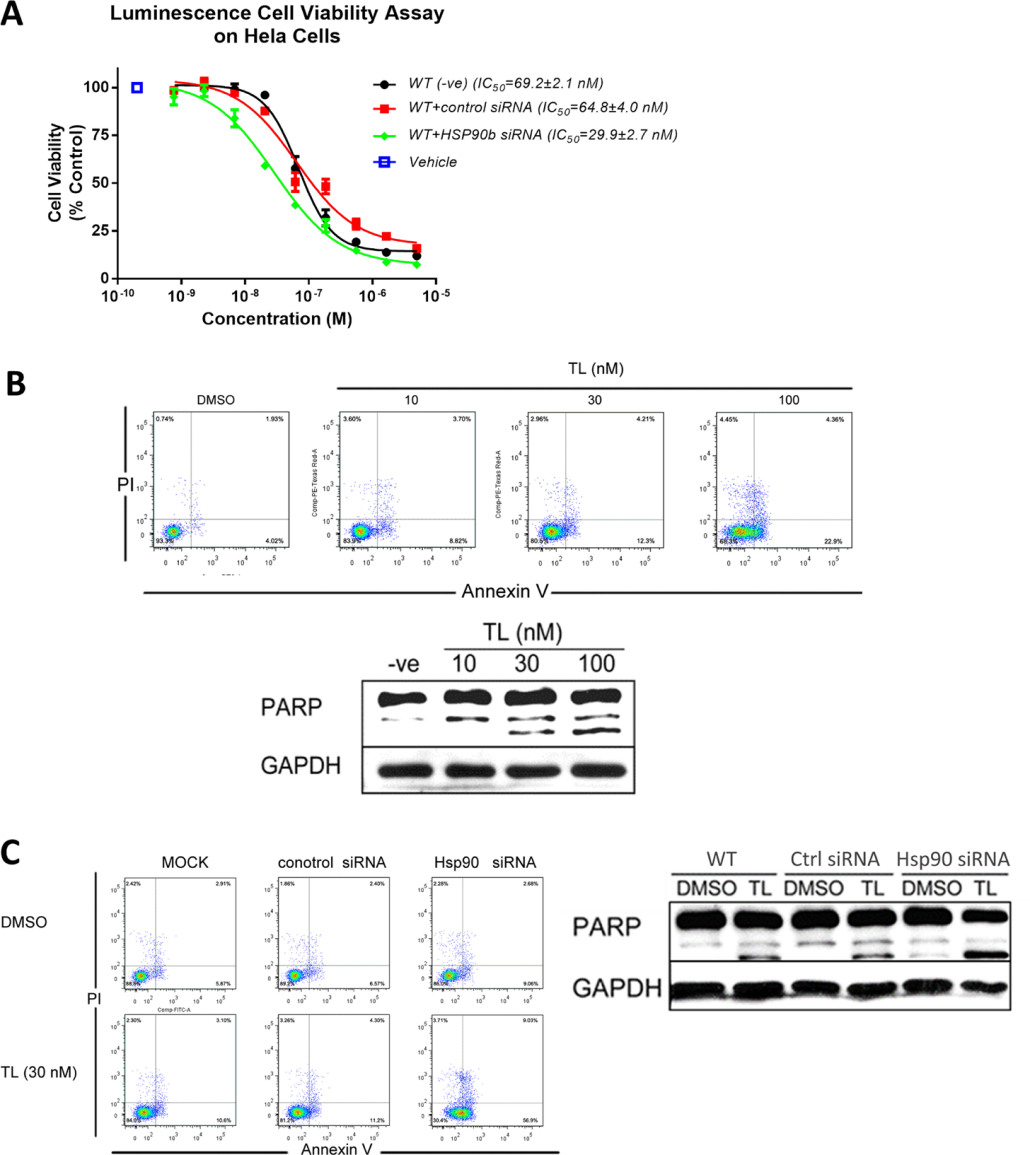
Triptolide triggers programmed cell death through HSP90β (**A**) TL exhibits cytotoxicity in HeLa cells. Luminescence cell proliferation assay was performed to determine cell viability of HeLa cells transfected with either control siRNA or HSP90β siRNA upon TL treatment for 24 hrs in different concentrations. IC_50_ from three independent experiment was determined. Data are presented as mean ± SD (*n* = 3). (**B**) TL treatment triggers apoptosis in HeLa cells. Annexin V/PI analysis (upper) of apoptotic HeLa cells upon treatment of different concentrations of TL for 24 hrs. Western Blot analyses (lower) of cleavage PARP of HeLa cells upon 24 hrs’ TL treatment. Representative image from three independent experiment was shown. (**C**) Knockdown of HSP90β sensitizes TL-triggered apoptosis in HeLa cells. Annexin V/PI analysis (left) of apoptotic cells and Western Blot (right) analyses of cleavage PARP of HeLa cells transfected with control siRNA or HSP90β siRNA in the presence and absence of TL for 24 hrs. Representative image from three independent experiment was shown.

Previous studies have shown that TL exhibits high cytotoxicity in various cell lines [36, 37]. We next investigated whether TL-triggered programmed cell death could be HSP90β related. Viability assay (Figure 4A) showed that knockdown of HSP90β potentiated TL cytotoxicity in HeLa cells as IC_50_ of TL in HSP90β knockdown cells (29.9 nM) was half of that in control siRNA transfected cells (64.8 nM). Flow cytometry analysis (Figure 4B, upper panel) showed that TL treatment increased both early and late apoptotic cells in HeLa cells in a dosage dependent manner (Early apoptotic cells: control 4.02%, 10 nM TL 8.82%, 30 nM TL 12.3% and 100 nM TL 22.9%; late apoptotic cells: control 1.93%, 10 nM TL 3.7%, 30 nM TL 4.21% and 100 nM TL 4.36%). WB analysis further showed that TL could induce PARP cleavage in a dosage dependent manner (Figure 4B, lower panel) which supported our findings on cellular apoptosis in flow cytometry analysis. Next, we setup to find out whether TL-induced apoptosis is HSP90β dependent. Flow cytometry analysis (Figure 4C, left panel) revealed that knockdown of HSP90β could potentiate the induction of early and late apoptotic cells triggered by TL treatment when compared with TL-treated control siRNA transfected cells in which a slight induction of apoptotic cells was detected upon 30 nM TL treatment. (Early apoptotic cells: control siRNA 6.57%, TL+ control siRNA 11.2%, TL+ HSP90siRNA 56.9%; late apoptotic cells: control siRNA 2.4%, TL+ control siRNA 4.3%, TL+ HSP90siRNA 9.03%). Conformed to our results on cellular apoptosis, knockdown of HSP90β alone also increased cleaved PARP while TL treatment further increased PARP cleavage (Figure 4C, right panel). Taken together, silencing of HSP90β sensitizes and increases TL's toxicity indicating that TL-induced cytotoxicity is through inhibiting HSP90β.

### TL induces site-specific phosphorylation of HSP90β in early phase

**Figure 5:**
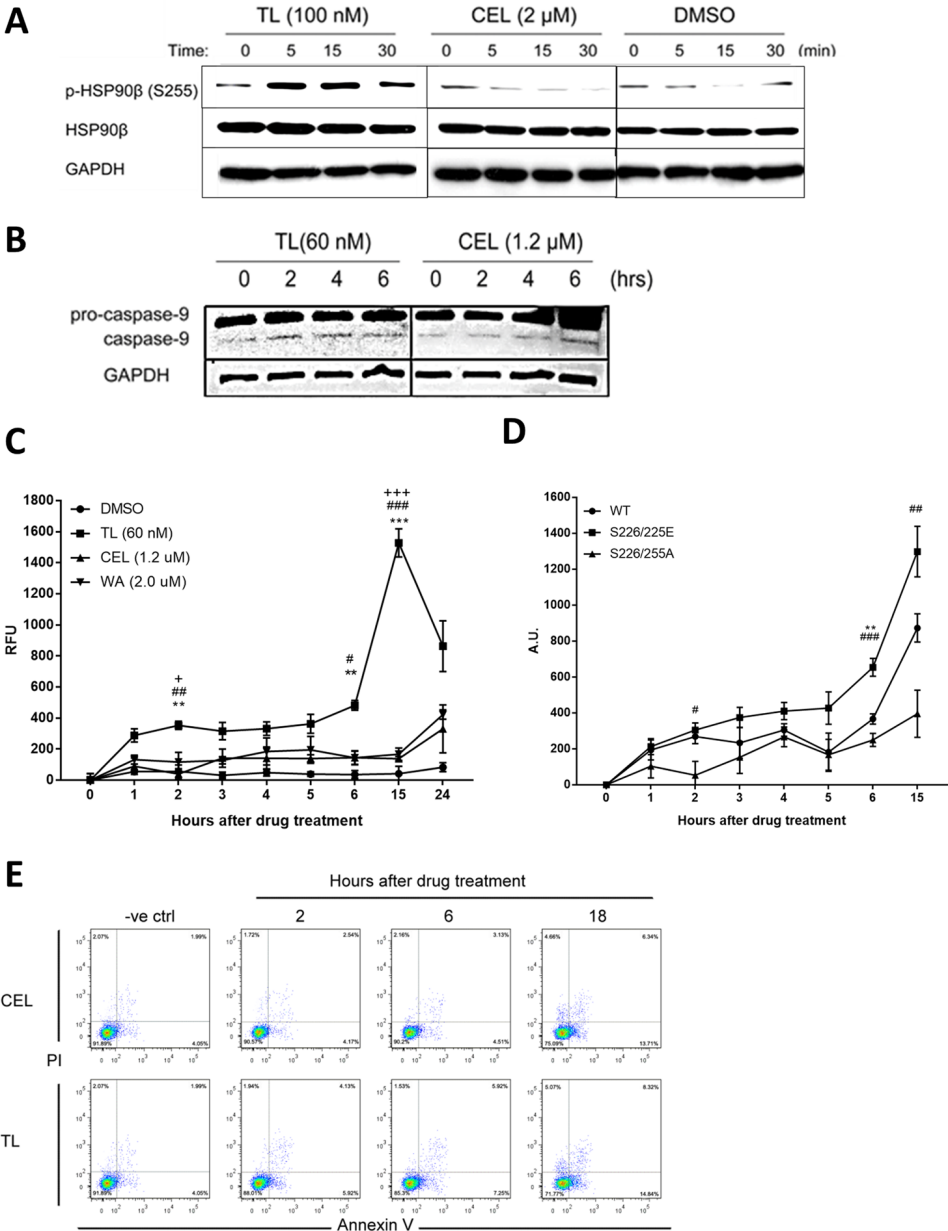
Phosphorylation of HSP90β is induced by TL in early phase (**A**) TL induces site-specific phosphorylation of HSP90β within 5 minutes of drug treatment. Western blot analysis of phosphorylation of HSP90β in HeLa cells treated with TL and CEL for 0, 5, 15 and 30 min. Representative image from three independent experiment was shown. (**B**) Western blot analysis of caspase-9 in TL/CEL-treated HeLa cells for 0, 2, 4 and 6 hrs. Representative image from three independent experiment was shown. (**C**) TL activates caspase-3 activity in the early phase. Caspase-3 activity was detected for HeLa cells upon TL, CEL and WA treatment over a time course from 0 to 24 hrs. At indicated time-points, cell lysate was prepared and incubated with assay buffer for at 37°C for 1 hr. Fluorescence was detected with an excitation at 405 nM and emission at 465 nM. ^**^P < 0.01; ^***^P < 0.001 DMSO versus TL. ^##^*P* < 0.01; ^###^*P* < 0.001 TL versus CEL. ^+^*P* < 0.05; ^+++^*P* < 0.001 TL versus WA. Data are presented as mean ± SD (*n* = 3). (**D**) Hypophosphorylation of HSP90β prevents TL mediated early activation of caspase-3 in HeLa cells. Caspase-3 activity assay was performed in HeLa cells transfected with different phosphorylation mutants of HSP90β. Data are presented as mean ± SD (*n* = 3). ^**^P < 0.01 WT versus S226/225E. ^#^*P* < 0.05; ^##^*P* < 0.01; ^###^*P* < 0.001 S226/225E versus S226/225A. (**E**) TL induces cell apoptosis in a shorter time-frame when compared to those upon CEL treatment. Annexin V/PI analysis of apoptotic HeLa cells upon TL and CEL for 2, 6 and 18 hrs.

So far, we have identified that TL inhibiting ATPase and chaperone activity and modifying the middle domain of HSP90β to disrupt its interaction with co-chaperone CDC37 are the long-term effects of the drug in mediating apoptosis. In asking whether any drug could have short-effect on the regulation of HSP90β in relation to early apoptosis-trigger, we could not find such drug in the literature but serine 226 and serine 255 of HSP90β were reported to be co-regulated to be involved in cytochrome C-mediated apoptosome activation [38]. We then examined whether TL could have a short-term impact on the phosphorylation of serine 255 of HSP90β. Time-course WB analysis (Figure 5A) showed that TL treatment of HeLa cells led to rapid phosphorylation of HSP90β within 5 minutes, then the induction slowly diminished after 30 minutes. In contrast, although CEL shares some similar long-term inhibition mechanisms of HSP90β as TL does, it did not induce the phosphorylation of HSP90β in the same manner as TL. Phosphorylation of serine 226 and 255 has been identified to facilitate apoptosome formation, a prerequisite for cellular apoptosis [38]. We then examined other early apoptotic markers, and cleaved caspase-9/3 is considered apoptotic marker for cells committed to programmed cell death. Triggered by the HSP90β phosphorylation induction, WB analysis showed that TL treatment caused a marked increase in the cleaved caspase-9 levels two hours upon treatment. Due to the lack of HSP90β phosphorylation induction, only marginal increase in the cleaved caspase-9 was observed four to six hours after CEL treatment (Figure 5B). Activated caspase-9 subsequently cleaves procaspase-3 to the activated form. We then examined the effects of TL on caspase-3 activity, the immediate downstream target of apoptosome. Due to the HSP90β phosphorylation induction and subsequent cleavage of caspase-9, TL induced caspase-3 activity significantly in HeLa cells one to two hour post treatment while caspase-3 activity was significantly and dramatically increased 15 hours post treatment, implying a bi-phasic activation of caspase-3 by TL (Figure 5C). As a result of the lack of HSP90β phosphorylation induction, CEL and WA only exhibited mono-phasic and modest activation of caspase-3 activity within 15 hours post treatment and the activation only started to rise after 24 hours post treatment. To further examine TL-induced early apoptosome activation through phosphorylation on HSP90β, HeLa cells were transfected with different HSP90β phosphorylation mutants (S226/255A and S226/255E) and tested for caspase-3 activity in the first 15 hours, a point where TL induced the highest activation of caspase-3 activity. Transfection of HeLa cells with HSP90β^S226/255A^ significantly delayed TL-induced caspase-3 early activation while transfection of HSP90β^WT^ and HSP90β^S226/255E^ did not significantly affect the activation of caspase-3 in the early phase (Figure 4D). Moreover, cells transfected with phosphorylation-mimicking mutant also potentiated the activation when compared to wild type. Flow cytometry analysis (Figure 5E) revealed that TL increased early apoptotic cells as early as 2 hours post treatment (from negative control: 4.05% to 2H: 5.92%) and apoptotic cell population increased afterwards (6H: 7.25%; 18H: 14.84%). In contrast, CEL could not increase percentages of apoptotic cells until 18 hours post treatment (from negative control: 4.05%; 2H: 4.17%; 6H: 4.51% to 18H: 13.71%), consistent with the results on the caspase-3 activity assay. To conclude, TL exhibits a distinct property that is different from other HSP90β inhibitors by triggering phosphorylation of HSP90β and subsequent activations in the early phase of drug treatment.

## DISCUSSION

HSP90β regulates multiple cellular pathways along with its co-chaperones which allows HSP90β appeared to be a primer target for drug development. Most HSP90β inhibitors shutdown HSP90β ATPase activity by targeting the ATP binding pocket of HSP90β at the N-terminal or the putative ATP binding site at the C-terminal. For instance, in two independent studies, Sun's group identified both CEL and WA bind to cysteine rich C-terminal of HSP90β [39, 40]. Although cysteine is one of the less abundant naturally occurring amino acids in proteins, cysteines especially those exposed on protein surface have often been found to crucial to protein function. Cysteine can impact diverse functions ranging from regulating protein-protein interaction to catalyzing enzymatic reaction, and reactive cysteines of a number of specific proteins become targets for drug development. A recent study revealed reactive cysteines on HSP90β through quantitative profiling under a chemical proteomics approach [41] and reported that cysteines 366 and 590 were the top two reactive cysteines among all other reactive cysteines identified in HSP90β.

The chaperone activity of HSP90β is predominantly regulated by its ATPase activity. Abolishing the ATPase activity of HSP90β adversely affects its dimerization of N-terminal domain resulting in loss of chaperone activity. Kupchan and coworkers [42] reported that TL modifies cysteine through alkylation of an epoxide group. In our current study, TL exhibits inhibitory effect on the ATPase activity of HSP90β as well as the ATP binding which is distinct from CEL which only inhibits ATPase activity. ATPase activity assay and ATP binding assay conducted on two HSP90β cysteine mutants revealed that TL's inhibition of ATPase activity is through preventing ATP binding and could not rule out the possible involvement of cys590 in TL-mediated inhibition. Martínez-Ruiz A and colleagues previously identified cysteine 590 on HSP90β or cysteine 597 on HSP90α1 being susceptible to nitrosylation [43], and nitrosylation on such cysteines decreases ATPase activity of HSP90β. Recent study conducted by Retzlaff suggested that ATPase activity of HSP90β is regulated by a C-terminal regulatory site through influencing its C-terminal dimerization [44]. Switching Alanine 577 in yHSP90 (counterpart of cysteine 590 in HSP90β) to Isoleucine led to stabilization of intra-molecular β-sheet at C-terminal of HSP90β while mutation of alanine 577 into asparagine increased C-terminal dissociation constant resulting a less stable HSP90β dimer. Potentially, modification of cysteine 590 by TL could possibly have the same effect as a β-sheet destabilizer at HSP90β C-terminal resulting in a decrease of ATPase activity. Furthermore, TL treatment suppresses HSP90β chaperone activity which was verified by luciferase refolding assay which is a likely consequence of inhibition of its ATPase activity. Our findings provide evidence for HSP90β's ATPase inhibition via ATP binding and subsequently inhibiting its chaperone activity of this cysteine reacting HSP90β inhibitor.

We then examined the downstream events of HSP90β chaperone inhibition. We found that TL decreases client protein levels of Raf-1 without altering its expression in HeLa cells. Study on identification of XPB as one of the targets of TL [34] suggested that TL has a universal transcriptional suppression effect through inhibiting RNA polymerase TFIIH activity. Surprisingly, TL does not specifically downregulate the transcription of HSP90β client proteins in our study. Besides, our data suggests that such suppression has no prominent effect on the HSP90β chaperone machinery. Our data further showed that TL has no significant change on expression levels at both mRNA and protein levels of HSP90β, its client proteins and co-chaperone such as IKK or CDC37 respectively.

CDC37 is a co-chaperone of HSP90β responsible for recruiting protein kinase clients of HSP90β-CDC37 complexes. Over the past decade, evidences including X-ray crystallography suggest that HSP90β interacts with CDC37 at its N-terminal domain [45]. Emerging evidences reveal CDC37 forms complex with HSP90β at both N-terminus and middle domains otherwise [18]. As both WA and CEL disrupt the HSP90β-CDC37 complexes through the N-terminal domain [40, 46], there is a growing interest to examine whether any drug could disrupts the HSP90β-CDC37 complexes through the middle domain. Nevertheless, dissociation of HSP90β-CDC37 complexes results in degradation of premature protein kinase clients. Pulldown assay using various HSP90β mutants and recombinant CDC37 revealed that mutation of cysteine 366 has no effect on HSP90β-CDC37 interaction itself while such mutation prevents TL-mediated destabilization of the HSP90β-CDC37 complexes. This observation is consistent with the fact that besides HSP90β interfaces with CDC37 at its N-terminal domain, a change in the middle domain could have an effect on the interaction. Our data showed that TL hinders the interaction between HSP90β and CDC37 by altering HSP90β micro-environment in the middle domain containing Cys366. Furthermore, we also confirmed that incubation of TL with CDC37 alone would have no effect on the interaction between HSP90β and CDC37, indicating that the disruption of the interaction mediated by TL is dependent on HSP90β.

Cell cycle progression is tightly fine-tuned by a group of CDCs and CDKs on discrete checking points inside cells. It has been reported that TL alters cell cycle profiling in a cell type dependent manner. It can be further explained by TL's widely different IC_50_ values and distinct cellular targets in different cell types. Dysfunction of HSP90β chaperone machinery by TL results in G1/G0 cell cycle arrest in HeLa cells. The consequence of the disruption of HSP90β-CDC37 complexes by TL is reflected by the decrease in client protein kinase CDK4 protein levels as well as the phosphorylation of Rb. Knockdown of HSP90β significantly sensitizes the cell cycle arrest effect triggered by TL thereby indicating that induction of cell cycle arrest by TL in HeLa cells is HSP90β-dependent. It is known that HSP90β machinery is responsible for maintaining the conformation, stability and function of many oncogenic client proteins in cancer cells [7, 47]. In addition to cell cycle arrest-induced apoptosis, degradation of HSP90β pro-survival client proteins triggers programmed cell death. There exists a high correlation between the abundance of HSP90β and TL induced-apoptosis. Knockdown of HSP90β dramatically enhanced TL's cytotoxicity verified by various apoptotic markers. Silencing of HSP90β caused an approximately 2-fold decrease of TL's IC_50_ in HeLa cells. It is possible that TL exerts most of its cytotoxic effect by inhibiting HSP90β; therefore, silencing of HSP90β is essentially the same as inhibiting HSP90β by TL. As a result, no drastic fold increase in potency was observed, and the 2-fold decrease was due to more silenced HSP90β as compared to TL-inhibited HSP90β.

In our study, silencing of HSP90β, expressed in higher abundance in cancer cells, shifts the IC_50_ to the left in HeLa cells. This result demonstrates that HSP90β mediates TL's effect as it takes lower effective concentration of TL to inhibit HSP90β in lower abundance in HSP90β knockdown cells. As shown in Figure 4A, at the lowest concentration of TL whereas the effect of TL is negligible, silencing of HSP90β has no effect on the cellular proliferation. This concludes that a 60% knockdown of HSP90β is probably sufficient to maintain cellular viability. The difference observed at higher concentration of TL is a direct reflection of TL-mediated inhibition of HSP90β. Although the molecular concentration of HSP90β in cancer cells is in μM range, TL, a nM drug, is only targeting functional HSP90β to exhibit its effect. Taken together, our study showed that HSP90β is potentially one of the few major targets of TL. TL's inhibition of HSP90β is upstream and transient making HSP90β a cancer selective and desirable target.

Kurokawa and colleagues reported that phosphorylation on serine 226 and 255 on HSP90β impedes inhibitory effect of HSP90β in relation to apoptosome formation [38]. Apart from the long-term effect of TL, CEL and WA on the inhibition of HSP90β to drive apoptosis, activation of caspase-3 activity, the immediate target of apoptosome, suggests a bi-phasic activation of caspase-3 triggered by TL. However, CEL and WA, in consistent with their long term effects on apoptotic induction, only exhibit a monophasic activation of caspase-3 in the long duration of drug treatment. Our results further indicate that only TL could induce caspase-3 activation at 1–2 hours after treatment which closely resembles the activation of caspase-3 by WT and phosphorylation-mimicking mutant of HSP90β in the first 1–2 hours of the early treatment. This early activation was not observed with the phosphorylation-blocking mutant could only suggest that the site-specific phosphorylation is responsible for the early phase but would not significantly affect the late phase activation of caspase-3 during the drug treatment. The observation that TL induces caspase-3 activation in the presence of WT HSP90β to a similar level that is the same as the phosphorylation-mimicking mutant strongly suggests that TL-mediated site-specific phosphorylation of HSP90β is responsible for the early activation of caspase-3. Flow cytometry data further confirmed that HeLa cells exhibit a much faster response to TL than CEL to induce early apoptotic cells. We first report a drug that is capable of inhibiting HSP90β by inducing its site-specific phosphorylation to trigger early apoptotic response.

In conclusion, we demonstrate that TL is a novel middle domain HSP90β inhibitor with a bi-phasic multi-mechanistic inhibition of HSP90β working in concert to trigger programmed cell death in cancer cells. TL inhibits ATPase activity of HSP90β by preventing ATP binding and decreasing overall HSP90β chaperone activity. In the meanwhile, TL disrupts HSP90β-CDC37 interaction through the middle domain cys366 resulting in kinase client protein degradation and cell cycle arrest. In contrast to other HSP90β inhibitors, TL exhibits a unique bi-phasic, in both short and long term, inhibition of HSP90β activity by inducing site-specific phosphorylation which offers a faster and more effective inhibition of HSP90β.

## MATERIALS AND METHODS

### Cells and reagents

Dulbecco's Modified Eagle Medium (DMEM), Fetal Bovine Serum (FBS), Penicillin/streptomycin (P/S) for cell culture, blocking solution for Western blot, MANT-ADP for ATPase binding assay, Opti-MEM medium, siRNA (HSP90β, scramble), lipofectamin 2000, lipofectamine RNAi Max for transfection, qRT-PCR kit were obtained from Life Technology. RIPA lysis buffer, ECL were purchased from Thermo; protease inhibitors and phosphor-stop were acquired from Roche. Strep-tactin superflow plus were purchased from Qiagen. Bradford protein assay kit was purchased from Bio-rad. High Throughput Colorimetric ATPase Assays kit were from Innova Biosciences. Caspase-3 activity assay kit was purchased from Cell Signaling. Apoptosis detection kit was purchased from BD. For antibodies, Raf-1, IKK-1/2, HSP90β, p-HSP90β, HSP70, CDC37, PPIA, p-MEK, MEK, p-ERK, ERK, GAPDH, PARP and Ub antibodies were purchased from Santa Cruz; Anti-flag antibodies was from Sigma; CDK4, p-Rb, Caspase-9, eEF2 and Strep-HRP antibodies were from Cell Signaling. All chemicals not listed above were purchased from Sigma-Aldrich.

### Protein expression and purification

BL21(DE3)_pET52b_HSP90β was inoculated in auto-induction medium and cultured for overnight at 1:200 ratio at 37°C. Bacteria culture was then incubated at 37°C until OD_600_ reached around 0.8 and subsequently further cultured for 18 hours at 18°C. Cells were lyzed by sonication in lysis buffer and centrifuged at 16,000 g for 20 minutes to remove cell debris. Supernatant were collected and passed through strep-tag column. Elutes were quantified and subjected to precision enzyme digestion to remove strep tag. Protein were further purified by passing through Q-column and followed by strep-tag column. In the last step, proteins were desalted and concentrated by 10K filtering units.

HEK-293T cells were cultured in DMEM and transfected with plasmids with PEI-HCl in Opti-MEM medium. Cells were harvest 48 hours after transfection and lyzed. Subsequently, strep-beads were incubated with cell lysate at 4°C. After washed by PBST for three times, strep-beads bound proteins were eluted by elution buffer (PBS with 2 mM desthiobiotin).

### ATPase assay

ATPase activity assay was performed using High Throughput Colorimetric ATPase Assays kit. Bacterial expressed recombinant HSP90β proteins (WT and different mutants) were incubated with TL and different inhibitors at 4°C for 3 hours and then added to reaction mixture according to the manufacturer's protocol. After incubated for additional 1 hour at 37°C, absorbance at 650 nM were detected and analyzed using Beckman Coulter plate-reader.

### MANT-ADP releasing assay

Recombinant HSP90β (WT and different mutants) were incubated with TL and different inhibitors for 3 hours 4°C. MANT-ADP was then added to the reaction mixture. The reaction mixture was incubated for additional 1 hour at 37°C and analyzed using Beckman Coulter plate-reader with excitation at 370 nM and emission at 465 nM.

### Ni-NTA pulldown assay

Recombinant HSP90β protein was preincubated with TL and different inhibitors at 4°C for 3 hours in incubation buffer (20 mM Tris, 150 mM NaCl, 5 mM MgCl_2_, 1% Triton-X-100, 10% glycerol, pH = 7.5). In the meanwhile, His-tagged CDC37 was incubated Ni-NTA beads at 4°C for 1 hour. His-CDC37-Ni-NTA complex was then mixed with drug-treated HSP90β for additional 3 hours. Ni-NTA beads were washed with the incubation buffer, and then bound proteins were eluted by boiling in sample buffer. Elutes were then subjected to immunoblot analysis.

### Streptavidin pulldown assay

Cells were washed with ice-cold PBS and harvested in a immunoprecipitation (IP) lysis buffer (50 mM HEPES, 2 mM MgCl_2_, 150 mM NaCl, 20% glycerol, 0.5% NP-40, pH = 7.5 with protease inhibitor cocktail). Lysates were centrifuged at 13,000 *g* for 10 min. Supernatant was collected and incubated with streptavidin overnight at 4°C. Beads were then washed with ice-cold lysis buffer and bound proteins were eluted by boiling in sample buffer. Elutes were then subject to immunoblot analysis.

### Immunoprecipitation assay

Cells upon treatment were washed with ice-cold PBS and harvested in an immunoprecipitation (IP) lysis buffer (50 mM HEPES, 2 mM MgCl_2_, 150 mM NaCl, 20% glycerol, 0.5% NP-40, pH = 7.5 with protease inhibitor cocktail). Lysates were centrifuged at 13,000 *g* for 10 min. The supernatant was collected and then incubated with primary antibodies overnight at 4°C. Protein A/G agarose beads were then added to each sample and incubated at 4°C for 2 hours. Beads were then washed three times with ice-cold lysis buffer and bound proteins were eluted by boiling in sample buffer. Elutes were then subject to immunoblot analysis.

### Western blotting

Cells were lysed in ice-cold RIPA lysis buffer and protein samples were subjected to electrophoresis in 8–12% SDS-polyacrylamide gels. Proteins were then transferred to PVDF membrane, blocked with 5% milk in TBST or blocking solution. Membrane was incubated with primary antibodies. After washing with TBST, blots were then probed with specific secondary antibodies linked to horseradish peroxidase (HRP). After washing with TBST, signals were then detected by chemoluminescence system.

### Transfection in HeLa cells

Hela cells were seeded in 6-well plate and transfected. For HSP90β knockdown, cells were transfected with mixture of siRNA and Lipo-RNAi Max transfection reagent in opti-MEM. For transfection with HSP90β expression vectors, cells were transfected with mixture of plasmid and Lipofectamine 2000 in opti-MEM. Cells were then treated with TL and different inhibitors for 24 hours and subjected to biochemical analyses.

### Flow cytometry analysis on HeLa cell

For PI-Annexin V staining, cells were washed by PBS and resuspended in Annexin V binding buffer with FITC Annexin V and propidium iodide solution. Cells were then incubated at room temperature for 15 minutes and subjected to flow cytometry analysis. For cell cycle analysis, cells were washed by PBS and resuspended in PI staining solution (20 μg/mL propidium iodide, 0.5% RNase A, 0.05% triton-X-100 in PBS). Cells were then incubated for 15 minutes and then subjected to flow cytometry analysis.

### Luciferase refolding assay

Luciferase was dissolved in protein buffer (25 mM Tris, 8 mM MgSO_4_, 0.1 mM EDTA and 0.5 mg/mL BSA, 10% glycerol and 1% triton X-100, pH = 7.5) to 0.5 mg/mL. For heat denaturing, luciferase was heated at 41°C for 10 min. The luciferase reaction master mix was prepared by mixing reaction buffer (100 mM Tris, 10 mM Mg (OAc)_2_, 375 mM KCl, 15 mM ATP, and 25 mM creatine phosphate, pH = 7.5) with CPK, heat-denatured native luciferase in deionized water. Drugs were dissolved in working buffer (20 mM Tris, 75 mM KCl, pH = 7.5), and then added to luciferase reaction master mix. Rabbit reticulocyte lysate was added to the mixture and the mixture was incubated at 4°C for 3 hours. Luciferase activity was measured by adding reaction mixture to luciferase assay buffer and incubated at room temperature for 5 minutes. Luminescence signal was measured with integration time of 400 ms on Beckman Coulter plate-reader.

### Real-time PCR

Total RNA was extracted from HeLa cells by RNA extraction kit. The quantitative real-time RT-PCR was performed using the Superscript III Platinum SYBR Green One Step qRT-PCR kit according to the protocols in Applied Biosystem PCR Detection System. Primers used for qRT-PCR were from PrimerBank (http://pga.mgh.harvard.edu/primerbank/) and the qRT-PCR results were normalized against GAPDH.

### Cell proliferation assay

For cell proliferation assay, CellTiter-Glo^®^ Reagent was added to cells upon treatment for cell lysis at room temperature. Cells were then incubated for 10 minutes, and luminescent signal was detected by Beckman Coulter plate-reader with 400 ms integration time.

### Caspase-3 activity assay

Cells upon assay were washed with PBS and lyzed in cell lysis buffer. Cell lysate was then incubated with assay buffer for at 37°C for 1 hour in dark. Fluorescent signal was detected by Beckman Coulter plate-reader with an excitation at 405 nM and emission at 465 nM.

### Statistical analysis

All data were presented as mean ± standard deviation (SD). Statistical significance among treatment groups were determined by Student's *T*-test and one-way analysis of variance (ANOVA). ^*^p < 0.05; ^**^p < 0.01^***^; *p* < 0.001 indicate statistical significance.

## SUPPLEMENTARY MATERIALS FIGURES AND TABLES



## Abbreviations

ADP: Adenosine diphosphate
ATP: Adenosine triphosphate
BME: 2-Mercaptoethanol
CDC: Cell division cycle
CDK: Cyclin-dependent kinase 4
CEL: Celastrol
Cys: Cysteine
DMEM: Dulbecco's Modified Eagle Medium
DMSO: Dimethyl sulfoxide
FACS: Fluorescence-activated cell sorting
GAPDH: Glyceraldehyde 3-phosphate dehydrogenase
HOP: HSP70-HSP90 organizing protein
HRP: Horseradish peroxidase
HSF-1: Heat shock factor-1
HSP: Heat Shock Protein
IC_50_: Half Maximal Inhibitory Concentration
MANT-ADP: (2’-(or-3’)-O-(N-Methylanthraniloyl) Adenosine 5’-Diphosphate
NOV: Novobiocin
PAGE: Polyacrylamide gel electrophoresis
PBS: Phosphate buffered saline
PEI: Polyethylenimine
PI: Propidium iodine
RAD: Radicicol
Rb: retinoblastoma protein
RT-PCR: real-time polymerase chain reaction
SDS: Sodium dodecyl sulfate
siRNA: small interfering RNA
Strep: streptactin
TL: Triptolide
TPR: tetratricopeptide repeat
TwHf: Tripterygium wilfordii Hook f
Ub: ubiquitination
WA: Withaferin A
WT: wild type
XPB: Xeroderma Pigmentosum B.
