# Supplementary material for: Triptolide, a HSP90 middle domain inhibitor, induces apoptosis in triple manner
Source: Oncotarget. 2018 Apr 27;9(32):22301–15. doi: 10.18632/oncotarget.24737 (PMC5976465; doi:10.18632/oncotarget.24737)
Supplement: Supplementary file 1 [file oncotarget-09-22301-s001.pdf]

Triptolide, a HSP90 middle domain inhibitor, induces apoptosis in triple manner

SUPPLEMENTARY MATERIALS

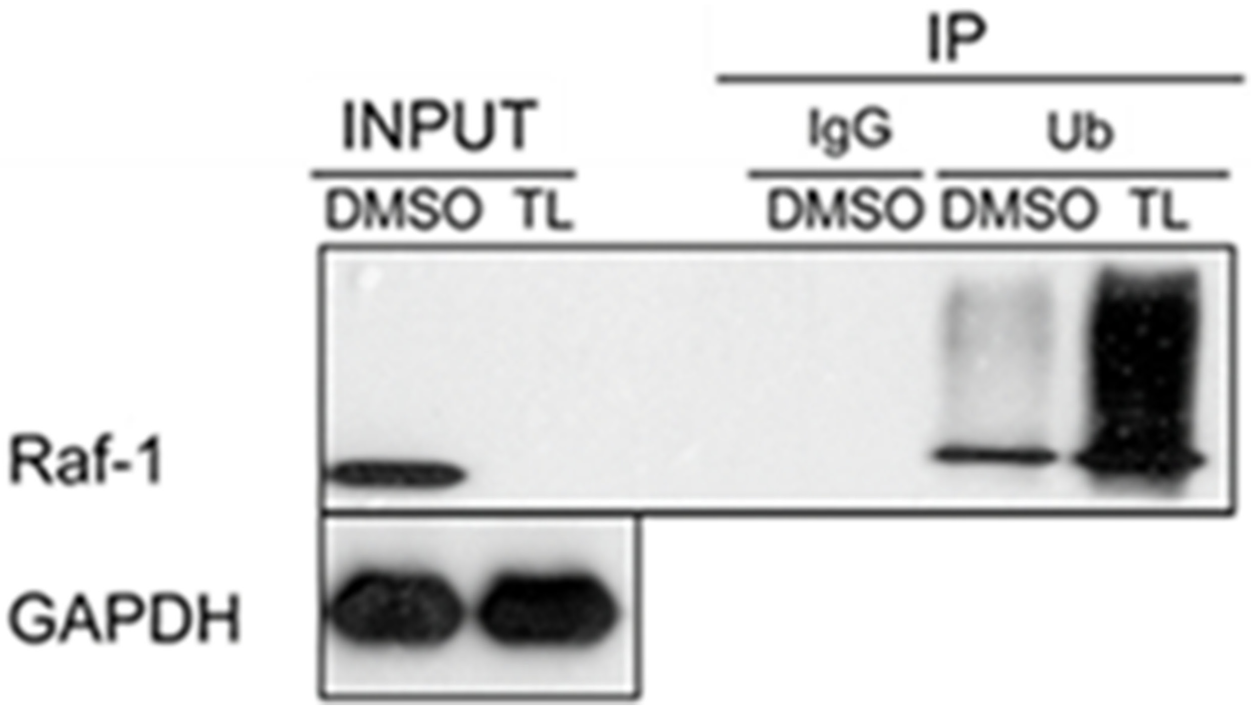

Supplementary Figure 1: TL treatment leads to degradation of specific HSP90β client protein Raf-1 through ubiquitination pathway. Immunoprecipitation of ubiqutinated proteins by anti-Ub antibodies and immunoblotted with Raf-1 antibodies.

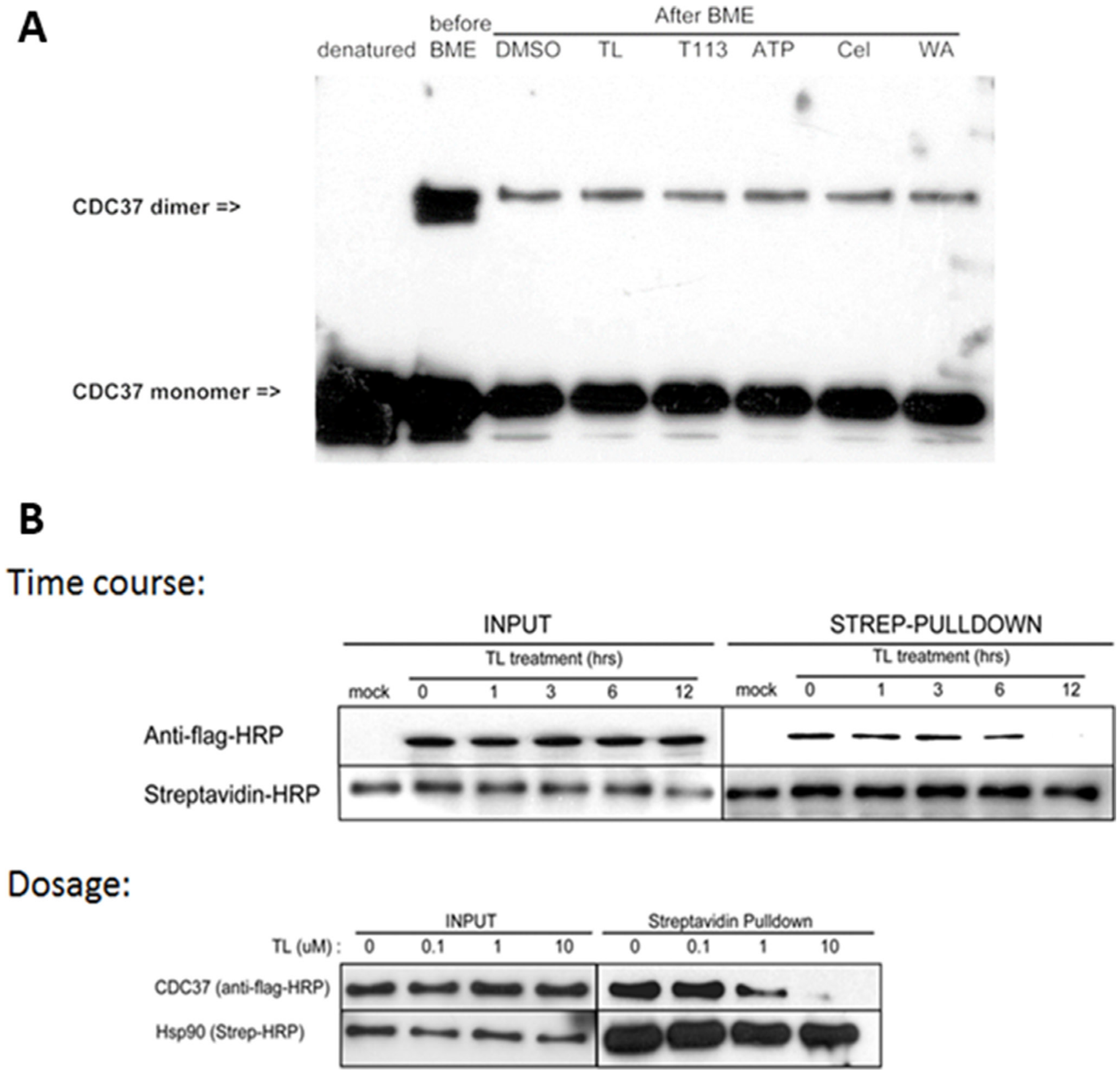

Supplementary Figure 2: (**A**) Non-reducing SDS-PAGE of dimeric status of CDC37 in HeLa cells upon treatment of TL and different inhibitors. (**B**) Co-immunoprecipitation assay on Hsp90β and CDC37 interaction upon treatment of TL for different time-points (time-dependent study, upper) and TL in different concentrations for 6 hrs (dosage-dependent study, lower).
